# The Impact of Dry Needling With Electrical Stimulation on Pain and Disability in Patients With Musculoskeletal Shoulder Pain: A Systematic Review and Meta-Analysis of Randomized Controlled Trials

**DOI:** 10.7759/cureus.41404

**Published:** 2023-07-05

**Authors:** Anthony N Baumann, Andrew Fiorentino, Caleb J Oleson, John Martin Leland

**Affiliations:** 1 Department of Rehabilitation Services, University Hospitals, Cleveland, USA; 2 College of Medicine, Northeast Ohio Medical University, Rootstown, USA; 3 Department of Orthopedic Surgery, University Hospitals, Cleveland, USA

**Keywords:** orthopedics, physical therapy, electrical stimulation, dry needling, musculoskeletal, shoulder pain

## Abstract

Musculoskeletal shoulder pain (MSP) is a common orthopedic condition frequently treated by orthopedic surgeons and physical therapists in an interdisciplinary manner. Dry needling with electrical stimulation (DNES) is an increasingly popular intervention used for the conservative treatment of MSP during physical therapy. To date, no systematic review and meta-analysis have examined the impact of DNES on outcomes in patients with MSP. This study aims to explore the effectiveness and safety of DNES in patients with MSP to improve patient outcomes. A systematic review and meta-analysis were conducted using PubMed, MEDLINE, CINAHL, and Web of Science from database inception to March 10, 2023. Inclusion criteria were studies with DNES as an intervention, recorded patient outcomes, and randomized controlled trials (RCTs) only. DNES with or without conventional physical therapy (CPT) was compared to CPT alone, which included interventions such as exercise, manual therapy, dry needling without electrical stimulation, and/or interferential current. A total of five RCTs were analyzed from 144 articles retrieved on the initial search. Included patients (n=342) had an average age of 48.75 ± 5.92 years, an average follow-up time of 3.40 ± 1.42 months, and 184 patients receiving DNES with or without CPT. Patients treated with DNES with or without CPT (n=163) had a frequency-weighted mean decrease in pain of 4.8 ± 1.4 points, whereas patients treated with CPT alone (n=158) had a frequency-weighted mean decrease in pain of 3.3 ± 2.2 points. For meta-analysis of pain outcomes (n=321 total patients), DNES with or without CPT improved pain by 1.40/10 points as compared to CPT alone with no significant difference between groups (p=0.203; Cohen’s d effect size (ES): 4.352; 95% CI: -2.343, 11.048). Patients treated with DNES with or without CPT (n=118) had a frequency-weighted mean decrease in disability of 34.7 ± 9.1 points. In contrast, patients treated with CPT alone (n=115) had a frequency-weighted decrease in disability of 20.1 ± 5.0 points. For meta-analysis of disability outcomes (n=233 total patients), DNES with or without CPT did not have a significant improvement in disability as compared to CPT alone (p=0.282; Cohen’s d ES: 0.543; 95% CI: -0.446, 1.532). No serious adverse effects were reported for patients treated with DNES with or without CPT or CPT alone. DNES with or without CPT may significantly improve pain and disability in patients with MSP. However, DNES with or without CPT does not provide statistically significant improvements in pain or disability compared to CPT alone. Furthermore, DNES appears to be a safe intervention for MSP.

## Introduction and background

Musculoskeletal shoulder pain (MSP) is a common condition frequently treated in a conservative manner by orthopedic surgeons and physical therapists via an interdisciplinary team approach [1-3]. MSP is a pathology that involves numerous etiologies, including adhesive capsulitis, rotator cuff tears, and hemiplegic shoulder pain after a stroke [4]. The literature indicates that MSP is the third most common reason to receive physical therapy, thus indicating the need for high-quality physical therapy interventions [5]. Common conservative interventions used in physical therapy for MSP include exercise, manual therapy, dry needling, and interferential current (IFC) [1-3]. Although many different physical therapy interventions exist for MSP, dry needling with electrical stimulation (DNES) is an increasingly popular intervention used to treat MSP during physical therapy to decrease patient pain and disability [2,4-9]. DNES is defined as a needling therapy in which electric current is conducted through small needles inserted into the skin and underlying musculature/soft tissue to improve patient pain and function and has many different names in the literature, such as electroacupuncture, DNES, and percutaneous electrolysis [2,4,6-8]. A meta-analysis related to this topic recently demonstrated that dry needling alone can lead to short-term benefits in pain and disability in patients with MSP [10]. However, it is unknown if the addition of electrical current via DNES can provide additional benefits in clinical outcomes for patients with MSP similar or greater to those seen with dry needling alone [3,10]. There has been recent interest in the impact of DNES on MSP as multiple randomized controlled trials (RCTs) have been published within the last five years [2,4-6,8]. To date, no systematic review and meta-analysis have examined the impact of DNES on outcomes in patients with MSP. However, dry needling alone has been explored extensively in the literature for many other musculoskeletal conditions via meta-analysis [11-15]. The purpose of this first-time systematic review and meta-analysis is to explore the efficacy and safety of DNES in patients with MSP to improve patient outcomes and provide information for appropriate referrals to facilitate interdisciplinary care.

## Review

Methods

Information Sources and Search Strategy

A systematic review and meta-analysis were conducted in this study by using PubMed, MEDLINE, CINAHL, and Web of Science from the database inception to March 10, 2023. The full search algorithm used in each database was ("dry needling" OR "acupuncture") AND (electric OR electrical) AND ("shoulder pain" OR "rotator cuff" OR "subacromial"). This study is in accordance with the most recent Preferred Reporting Items for Systematic Reviews and Meta-Analyses (PRISMA) guidelines [16]. This systematic review was not registered prior to manuscript completion. 

Inclusion and Exclusion Criteria

Inclusion criteria for this study were studies with DNES as an intervention, diagnosis of musculoskeletal shoulder pain, recorded patient outcomes, full-text articles, articles in English, and RCTs only. Exclusion criteria for this study were no interventions with DNES in the study, pain in the shoulder region unrelated to musculoskeletal shoulder pain (i.e., cervical radiculopathy), lower level of evidence studies, systematic reviews, meta-analyses, books, non-full text articles, and non-English articles.

Study Definitions

For the purposes of this study, DNES is defined as any form of dry needling or acupuncture that uses needle insertion into muscles and surrounding soft tissue along with the use of electrical stimulation provided by electrical current conducted through the needle. For this study, CPT is defined as numerous interventions, such as manual therapy, exercise, IFC, and other physical therapy interventions. For reported minimal clinically important difference (MCID), this study used 1.4/10 points for pain and 8 points for disability [3,17,18].

Selection Process

Article screening began with the use of the Rayyan software, which has been used elsewhere in the literature for systematic reviews and meta-analyses [19]. First, duplicate articles were removed manually, which was then followed by article screening by title and abstract per the inclusion and exclusion criteria. Articles were then screened by full text for final inclusion in this study. 

Data Extraction

Two authors performed data extraction for this study. Data collected included first author, year of publication, type of study (RCTs), information on DNES or control group, average patient age, number of included patients, pain outcomes via a 0-10 scale, disability outcomes via the Shoulder Pain and Disability Index (SPADI), adverse events and/or complications, and follow-up time.

Bias and Quality Assessment

This study's bias and quality assessment was completed via the Physiotherapy Evidence Database (PEDro) scale [20]. The PEDro scale is reported on a 0-10 scale, with each criterion worth 0-10 points. For grading, the PEDro scores are interpreted as poor (0-3), fair (4-5), good (6-8), and excellent (9-10) in terms of study quality.

Synthesis Methods

A random-effects continuous model meta-analysis using Cohen's d was performed for pain (0-10 scale) and disability (SPADI). The random-effects model was used due to the heterogeneity of the interventions of the included articles and precedent in the literature with similar meta-analyses for physical therapy interventions [11,21]. Unstandardized mean difference (UMD) was used for effect size only to calculate the difference in improvement between the two groups for pain and was not used to calculate significance levels. UMD was not calculated for SPADI as disability was recorded differently in the included articles (out of 100 and 130 points). The SPSS version 29.0 (IBM Corp, Armonk, NY, US) was used for the statistical analysis of this study. Frequency-weighted means were used for patient demographics and descriptive data.

Reporting Bias Assessment

This study did not assess for the existence of publication bias via funnel plot asymmetry due to the small sample size of five included articles [22,23].

Certainty Assessment

This study utilized the Grading of Recommendations, Assessment, Development, and Evaluation (GRADE) approach to assess the certainty of evidence [24]. The certainty of the evidence was rated as high, moderate, low, or very low for each included analysis based on the study design, risk of bias, heterogeneity, indirectness, and publication bias of the evidence [24].

Results

Study Selection

**Figure 1:**
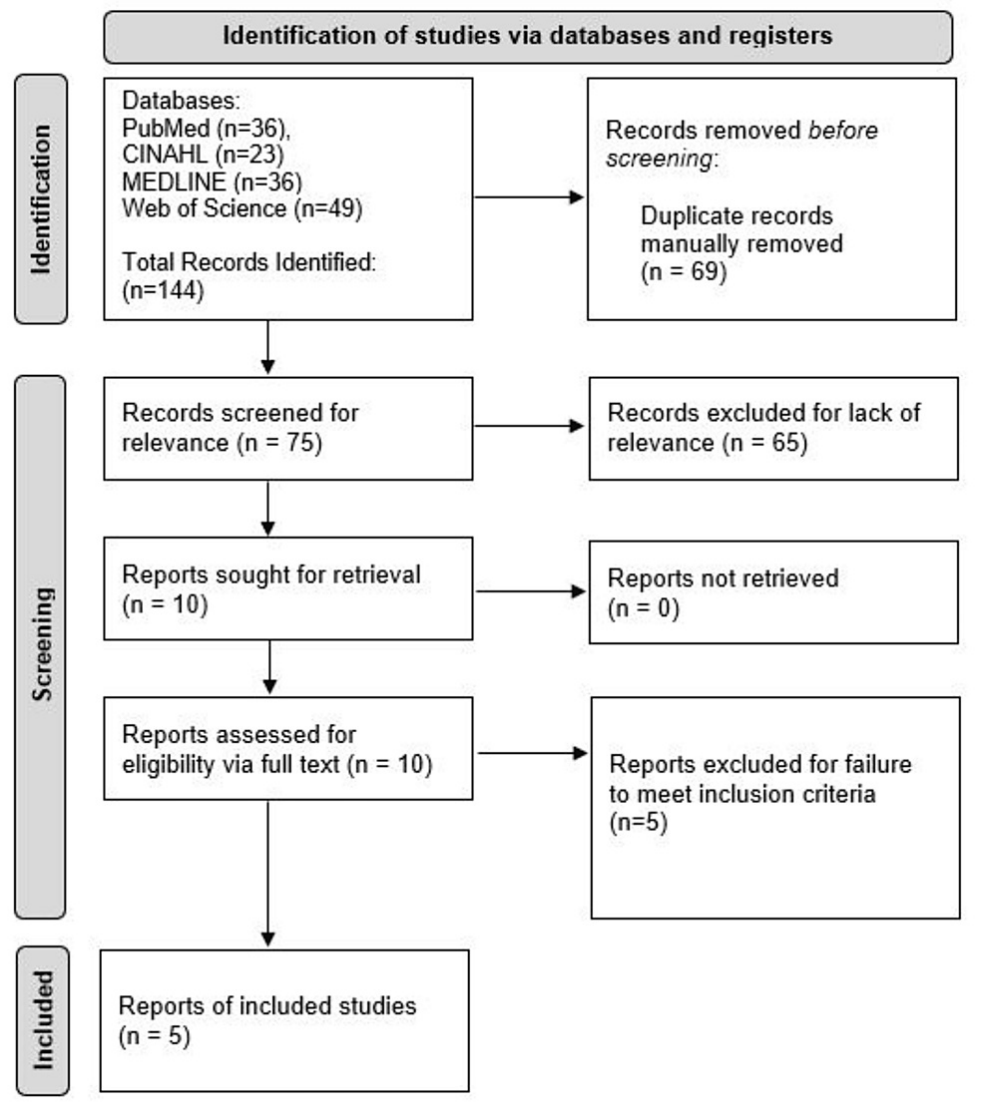
The Preferred Reporting Items for Systematic Reviews and Meta-Analyses (PRISMA) diagram outlining the search process from initial search to final article inclusion.

A total of five RCTs were analyzed after meeting inclusion criteria from a total of 144 articles retrieved on the initial search [2,4-6,8]. Figure 1 below provides information on the systematic review process from the initial search to the final article inclusion.

Risk of Bias in Included Studies

Based on the PEDro scale for bias and quality, there was one included article with fair quality (20%), and four included articles with good quality (80%). None of the included articles had poor or excellent quality as determined by the PEDro scale. Table 1 below provides more information on bias and quality assessment for the included articles via the PEDro scale.

**Table 1:** Information on the bias and quality grading via the Physiotherapy Evidence Database (PEDro) scale. Information includes first author, year of publication, total PEDro score from 0-10 points, presence of eligibility criteria as specified by the PEDro scale, and components of the PEDro scale with scoring from 0-1 points. The five included articles were graded via the PEDro scale [2,4-6,8].

Author (year)	PEDro score	Eligibility criteria were specified	Random allocation	Allocation was concealed	Groups similar at baseline	Subject blinding	Therapist blinding	Assessor blinding	Less than 15% dropouts	Intention-to-treat analysis	Between-group statistical comparisons	Point measures and variability data
Dunning J et al. (2021) [2]	8	Yes	1	1	1	0	0	1	1	1	1	1
Eslamian F et al. (2020) [4]	7	Yes	1	1	1	0	0	1	1	0	1	1
de Miguel Valtierra L al. (2018) [5]	8	Yes	1	1	1	0	0	1	1	1	1	1
Shanmugam S et al. (2021) [6]	8	Yes	1	1	1	0	0	1	1	1	1	1
Lo MY et al. (2020) [8]	5	Yes	1	0	1	1	0	0	0	0	1	1

Study Characteristics and Patient Demographics

Included patients (n=342) had an average age of 48.75 ± 5.92 years with an average follow-up time of 3.40 ± 1.42 months and 184 patients receiving DNES with or without CPT. Table 2 below provides additional information on each of the five included articles. No serious adverse effects were reported for patients treated with DNES with or without CPT or CPT alone.

**Table 2:** Demographic and outcome data from the five included articles for this systematic review and meta-analysis. Data recorded includes first author, year of publication, group, diagnosis, information on dry needling with electrical stimulation, number of patients, average age, pre and post-pain, pre and post-disability (Shoulder Pain and Disability Index), adverse events/complications, and follow-up time. Five articles were included in this study [2,4-6,8]. RCT: Randomized controlled trial; DNES: Dry needling with electrical stimulation; SPADI: Shoulder Pain and Disability Index; IFC: Interferential current.

First author (year)	Type of study	Study group	Diagnosis	Type of DNES	Patients (n)	Average age (years)	Average pre-pain	Average post-pain	Average pre-SPADI	Average post-SPADI	Complications	Follow-up
Dunning J et al. (2021) [2]	RCT	Spinal thrust manipulation and electrical dry needling	Subacromial pain syndrome	Electrical dry needling (using a standardized protocol of 8 obligatory points)	73	46.2 ± 15.6	5.4 ± 1.4	1.4 ± 1.6	44.9 ± 14.6	9.9 ± 10.1	Thirty-seven patients assigned to the electric dry needling group (50.7%) experienced post-needling muscle soreness, and 15 (20.5%) experienced mild bruising (ecchymosis), which most commonly resolved spontaneously within 48 hours and 2-4 days, respectively. Two patients (2.7%) in the electric dry needling group experienced drowsiness, headache, or nausea, which spontaneously resolved within several hours.	3 months
		Non-thrust peripheral joint/soft tissue mobilization, exercise, and interferential current (control group)	Subacromial pain syndrome	-	72	41.8 ± 15.8	5.2 ± 1.6	3.3 ± 1.9	43.3 ± 16.2	26.1 ± 17.6	No adverse events were reported in the control group.	3 months
Eslamian F et al. (2020) [4]	RCT	Group A received conventional trainings with additional IFC with medium frequency of 4000 Hertz	Hemiplegic shoulder pain caused by ischemic stroke	-	20	57.55 ± 1.73	5.25 ± 0.63	3.65 ± 0.54	99.00 ± 5.69	79.65 ± 5.45	No adverse events including bleeding, infection or sustained pain related to electroacupuncture or IFC were seen in the present study.	1.25 months
		Group B received conventional training with additional electroacupuncture two times a week for a total of 10 sessions.	Hemiplegic shoulder pain caused by ischemic stroke	Electrical acupuncture	20	57.3 ± 3.71	6.90 ± 2.67	4.35 ± 0.7	112.3 ± 3.42	94.5 ± 4.09	No adverse events including bleeding, infection or sustained pain related to electroacupuncture or IFC were seen in the present study	1.25 months
de Miguel Valtierra L et al. (2018) [5]	RCT	Manual therapy and exercise	Subacromial pain syndrome	-	23	55.3 ± 11.1	6.8 ± 1.9	4.1 ± 3.4	57.6 ± 16.9	27.6 ± 17.1	No significant adverse events were reported.	6 months
		Manual therapy and exercise plus ultrasound-guided percutaneous electrolysis	Subacromial pain syndrome	The technique was applied using a device that produces modulated galvanic electricity through the negative electrode cathodic flow. The galvanic current is applied using acupuncture needles. This technique was performed through ultrasound under guidance.	25	54.9 ± 13.7	6.9 ± 1.6	1.5 ± 1.8	57.4 ± 14.7	10.1 ± 6.5	Six (24%) patients within the percutaneous electrolysis group experienced muscle soreness after the first 2 treatments, which resolved spontaneously at 24 to 36 hours.	6 months
Shanmugam S (2021) [6]	RCT	Group 1: Intramuscular electrical stimulation combined with therapeutic exercises	Shoulder adhesive capsulitis	Intramuscular electrical stimulation - similar method of dry needling with additional intramuscular electrical stimulation using inverse electrode placement	45	47.82 ± 6.13	7.18 ± 0.94	0.42 ± 0.5	-	-	No serious adverse effects occurred during the three weeks of treatment. Non-serious adverse events include dry-needling induced soreness, severe pain during needling, profuse sweating, excessive post-needling pain.	3 months
		Group 2: Dry needling combined with therapeutic exercises	Shoulder adhesive capsulitis	-	43	45.26 ± 4.81	7.47 ± 0.7	0.63 ± 0.54	-	-	No serious adverse effects occurred during the three weeks of treatment. Non-serious adverse events include dry-needling induced soreness, severe pain during needling, profuse sweating, excessive post-needling pain.	3 months
Lo MY et al. (2020) [8]	RCT	True electroacupuncture group	Frozen shoulder syndrome	Stimulation occurred with an alternating frequency of 2-3 Hertz at a pulse duration of 100-400 for 20 minutes	11	60.8 ± 5.1	-	-	-	-	No significant adverse events were reported in either group.	6 months
		Sham electroacupuncture group	Frozen shoulder syndrome	-	10	58.3 ± 7.1	-	-	-	-	No significant adverse events were reported in either group.	6 months

Impact of Dry Needling with Electrical Stimulation on Pain Outcomes

**Figure 2:**
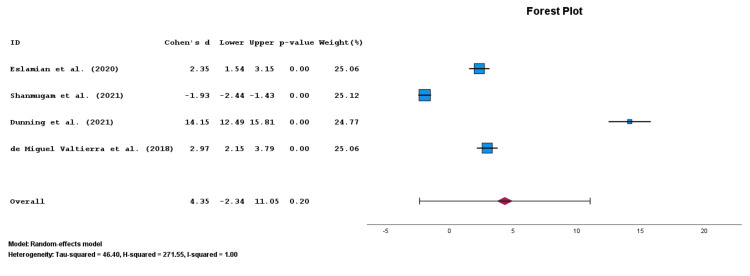
Forest plot for patients with musculoskeletal pain treated with dry needling with electrical stimulation (DNES) compared to conventional physical therapy (CPT). Four articles were included in this forest plot [2,4-6].

Patients treated with DNES with or without CPT (n=163) had a frequency-weighted mean decrease in pain of 4.8 ± 1.4 points, whereas patients treated with CPT alone (n=158) had a frequency-weighted mean decrease in pain of 3.3 ± 2.2 points. For meta-analysis of pain outcomes from four included articles (n=321 total patients), DNES with or without CPT (n=163) improved pain by 1.40/10 points via UMD as compared to CPT alone (n=158) with no significant difference between groups (p=0.203; Cohen's d ES: 4.352; 95% CIs: -2.343, 11.048; Figure 2) [2,4-6]. There was a high degree of heterogeneity (I2=1.00). Certainty via GRADE was judged to be "moderate" due to inconsistency [24]. All four articles used for the pain outcomes meta-analysis had a rating of "good" via PEDro for bias and quality assessment [2,4-6].

Impact of Dry Needling with Electrical Stimulation on Disability Outcomes

**Figure 3:**
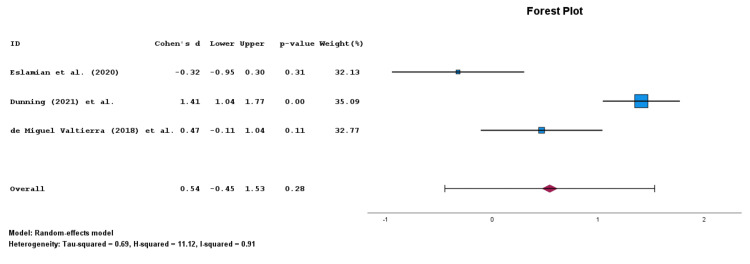
Forest plot for disability outcomes via the Shoulder Pain and Disability Index (SPADI) between patients treated with dry needling with electrical stimulation (DNES) as compared to conventional physical therapy (CPT) for musculoskeletal shoulder pain. Three articles were included in this forest plot [2,4,5].

Patients treated with DNES with or without CPT (n=118) had a frequency-weighted mean decrease in disability of 34.7 ± 9.1 points. In contrast, patients treated with CPT alone (n=115) had a frequency-weighted decrease in disability of 20.1 ± 5.0 points. For the meta-analysis of disability outcomes from three included articles (n=233 total patients), DNES with or without CPT (n=118) had no significant improvement in disability as compared to CPT alone (n=115) (p=0.282; Cohen's d ES: 0.543; 95% CIs: -0.446, 1.532; Figure 3) [2,4,5]. UMD was not calculated due to the difference in scoring for disability via the SPADI outcome measure. There was a high degree of heterogeneity (I2=0.91). Certainty assessment via GRADE was determined to be "moderate" due to inconsistency. All three articles in the disability outcomes meta-analysis had a "good" score via PEDro for the bias and quality assessment [2,4,5].

Discussion

This study represents the first systematic review and meta-analysis of the efficacy and safety of DNES for patients with MSP. Overall, this study found that DNES may provide clinically significant improvements in pain and disability in patients with MSP; however, these improvements are not statistically significant compared to interventions commonly used in CPT. Therefore, the findings of this study cautiously suggest that DNES can be a viable alternative to other interventions commonly used in CPT based on patient or clinician preference. Besides the clinical impact of DNES, this study also attempted to determine the safety of DNES. Despite almost 200 patients receiving treatment with DNES in this study, there were no cases of serious adverse events/complications, with all adverse events being minor and/or transient. Therefore, this study cautiously demonstrates that DNES can be a safe intervention when used with or without CPT. To be sure, there is heterogeneity in the diagnosis of MSP as there exist various overlapping etiologies within MSP, with myofascial trigger points (MTPs) being a common cause of MSP symptoms [4,6]. MTPs are a frequent target of conventional dry needling, with or without electrical stimulation. Dry needling alone has been shown to effectively reduce pain in a wide variety of conditions [6,10]. In terms of pathophysiology, dry needling can have numerous biochemical (increased endorphin levels) and neurophysiological (interruption of pain afferents via stimulation of large, myelinated nerve fibers) beneficial effects, especially when added to a physical therapy program [2,4]. Furthermore, electrical stimulation is frequently used for pain control in a non-invasive manner during physical therapy and is a common component of CPT [2,4]. This study aimed to determine if the combination of these two effective treatments, electrical stimulation and dry needling in the form of DNES, would lead to superior outcomes to these interventions alone. However, this was unfounded as there was no significant difference between DNES with or without CPT and CPT alone, indicating that the combination of these interventions did not produce additional meaningful improvements. Notably, the DNES protocols varied among the included articles, thus presenting a challenge for determining the true impact of DNES on clinical outcomes in patients with MSP. This difference may explain why DNES with or without CPT did not produce statistically significant improvement compared to CPT alone, although this remains to be elucidated. As the location of MTPs can vary in patients with shoulder pain, adding extra locations for inserting needles during DNES has been described in the literature [2]. Furthermore, one included study used insertion sites down the affected upper extremity to the hand [4]. While some included studies used manual palpation to determine needle insertion, others used ultrasound for proper needle insertion [2,4,5]. This heterogeneity in the application of DNES makes proper examination of the efficacy of DNES difficult; however, this difficulty is not unique to DNES as many other CPT interventions, such as exercise, face the same problems [1,2,11]. Although MCID values can vary to determine clinical significance and impact the study interpretation, a recent meta-analysis on dry needling used 1.4 for the 0-10 visual analog scale to determine clinical significance; therefore, this study sought to maintain the same threshold to determine clinical significance [3]. This MCID is also represented elsewhere in the literature for shoulder conditions [18]. There are additional limitations to this study that impact the interpretation of these results. To begin, CPT is a heterogeneous group of interventions, making comparisons between interventions difficult. A limitation of this study is that an included article used DNES and spinal thrust manipulation as an experimental group versus non-thrust peripheral joint manual techniques, exercise, and IFC for a control group [2]. This combination of different commonly used physical therapy interventions could explain the large ES for the article for pain improvement (Cohen's d ES=14.15), which likely influenced the large ES despite no statistical significance between the DNES and CPT groups. Although manual therapy combined with exercise can be part of an effective physical therapy program, the superiority of different manual techniques (thrust versus non-thrust) have yet to be solidified [2]. Another limitation is the relatively small sample size, as this systematic review and meta-analysis only involved five articles, despite a relatively low risk of bias (80% of articles received a "good" score on the PEDro scale) and a moderate certainty for both pain and disability outcomes via the GRADE scale. More RCTs are needed with better control groups to account for heterogeneity and a small sample size to solidify the role that DNES has in the conservative treatment of MSP. Further research should focus on comparisons between DNES and dry needling alone, as dry needling alone has been shown to improve outcomes in MSP [10].

## Conclusions

DNES with or without CPT may provide clinically significant improvements in pain and disability in patients with MSP. However, DNES with or without CPT does not provide statistically significant improvements in pain or disability compared to CPT alone based on a meta-analysis of five fair-to-good quality RCTs. Furthermore, DNES appears to be a safe intervention for MSP and could be a viable alternative to other interventions used in CPT. This study represents the first systematic review and meta-analysis of the effectiveness and safety of DNES for patients with shoulder pain. More research is needed on this topic as the relatively small sample size and heterogeneity of the included articles impact study interpretation and application.
